# A critical guideline for controlling monocyte-derived macrophages phenotypes

**DOI:** 10.3389/fimmu.2025.1694625

**Published:** 2026-02-23

**Authors:** Giorgia Moschetti, Doriana Oliveri, Valeria De Matteis, Marta Zaccaria, Diego Rondelli, Anna Griego, Edoardo Scarpa, Loris Rizzello

**Affiliations:** 1 Department of Pharmaceutical Sciences, University of Milan, Milan, Italy; 2 Infection Dynamics Laboratory, National Institute for Molecular Genetics (INGM), Milan, Italy; 3 Department of Experimental Medicine, University of Salento, Lecce, Italy

**Keywords:** macrophage ontogeny, macrophage plasticity and polarization, monocyte-derived macrophages (MDMs), tissue resident macrophages, tissue-specific microenvironment

## Abstract

Macrophages (Mϕ) are an extremely heterogeneous and rapidly adapting set of innate immune cells that are scattered throughout all tissues in humans from mid-gestation onwards. Their original definition as key players in phagocytosis and defense against pathogens is too restrictive nowadays, as Mϕ are central to tissue homeostasis, repair, and complex immune regulations involving adaptive immunity. The Mϕ exhibit different ontogenies, originating from either embryonic progenitors or bone marrow, and their fate is shaped by tissue-specific microenvironments, which determine their adaptive phenotypes. This results in functional flexibility, exemplified by their ability to polarize into pro- (M1) or anti- (M2) inflammatory states in response to environmental cues. Such a dynamic process is critical for resolving infections, repairing tissue, and maintaining immune balance. Dysregulated Mϕ polarization is indeed implicated in various pathologies, including chronic inflammation, cancer, and fibrosis. Despite their importance, the study of tissue-resident Mϕ is still limited by technical challenges related to their isolation, maintenance, and donor variability. As an alternative, monocyte-derived macrophages (MDMs) represent an easier *in vitro* system to model human Mϕ biology under controlled conditions. However, MDMs differ from tissue-resident Mϕ in their developmental origin and functional specialization. This review outlines the key principles and limitations of MDM-based models, discusses commonly used differentiation protocols, and proposes methodological strategies to enhance reproducibility and physiological relevance in macrophage research.

## 1 Introduction

Macrophages (Mϕ) are key immune cells that are ubiquitously distributed across all tissues of the human body, from mid-gestation throughout the entire individual lifespan (1). These cells play key roles in promoting tissue homeostasis by responding to exo- as well as endogenous stimuli and insults. Their role extends far beyond acting as phagocytes in defense against microbes or as removers of dead and senescent cells, as they also play important trophic, regulatory, and repair functions (2). Moreover, macrophages contribute to the orchestration of both innate and adaptive immune responses through the complex interaction with other immune cells (both innate and adaptive immune system) (3–5).

During organogenesis, Mϕ can originate either from embryonic yolk sac or fetal liver precursors, establishing self-maintaining resident populations in adult tissues, such as microglia in the central nervous system, Kupffer cells in the liver, and alveolar macrophages in the lungs (1, 2). Resident Mϕ support tissue homeostasis by clearing apoptotic cells and debris, as well as performing specialized organ-specific functions. For example, splenic red pulp macrophages (RPMs) are critical for heme and iron recycling, thereby maintaining systemic iron homeostasis and supporting efficient erythrocyte turnover (6). After birth, bone marrow-derived monocytes replenish Mϕ in high-turnover tissues like the gut, and they are also recruited to the site of injury, infection, and inflammation, where they contribute to tissue repair, fibrosis, and angiogenesis (7). Thus, in every tissue, Mϕ populations consist of both embryonic-derived residents and infiltrating monocytes, leading to significant phenotypic diversity based on their origin and environmental cues.

Mϕ can be broadly categorized into two main types based on their ontogeny: tissue-resident macrophages (TRMs), which are established during embryogenesis, and monocyte-derived macrophages (MDMs), which originate from circulating bone marrow–derived monocytes. Embryonic-derived TRMs, such as microglia in the brain and Kupffer cells in the liver, are typically long-lived, self-renewing, and highly specialized to meet the structural and functional demands of their respective tissue niches. In contrast, MDMs are continuously replenished from the bone marrow, particularly in high-turnover tissues such as the gut, and are rapidly recruited in response to infection, inflammation, or injury. Upon tissue entry, monocytes undergo differentiation and polarization in response to local environmental cues, including extracellular matrix components and cytokines secreted by resident cells. For example, the successful engraftment and identity acquisition of monocyte-derived cells in the liver requires interactions with hepatocytes, endothelial cells, and hepatic stellate cells, in conjunction with signals such as TGF-β and desmosterol (8, 9).

With aging, embryonic-derived TRMs are progressively replaced by MDMs in various organs, including the heart, kidneys, and gut. Inflammatory insults can also drive the transient or permanent replacement of TRMs by infiltrating monocytes. Once integrated into the tissue, MDMs may adopt many features of resident macrophages by responding to local cytokines, growth factors, and cell–cell interactions. However, their capacity to fully replicate the phenotype and function of embryonic-derived TRMs is context-dependent and often constrained by the availability of spatial niches within the tissue microenvironment.

However, there are no definitive markers currently that reliably distinguish embryonic-derived TRMs from bone marrow–derived MDMs in human tissues, posing a major challenge for dissecting macrophage ontogeny in both health and disease (10).

The dual origin of macrophages, combined with an extremely high level of tissue-specific distribution and specialization, necessitates a high degree of Mϕ plasticity, allowing them to adapt dynamically to local microenvironments. Resident Mϕ display different functionalities depending on their tissue environment, reflecting their adaptation to specific organ needs. Microglia, for instance, are highly specialized in synaptic clipping and neuronal support in the brain (11, 12), while Kupffer cells regulate iron metabolism and detoxification in the liver (13, 14). Similarly, alveolar Mϕ in the lungs must balance defense mechanisms together with immune tolerance towards inhaled particles and commensal microbes (15, 16).

Mϕ are highly plastic immune cells capable of adopting distinct functional phenotypes in response to environmental signals, particularly during immune responses (17, 18). These cells have been historically classified into pro-inflammatory/classically activated (M1) and anti-inflammatory/alternatively activated (M2) phenotypes (19). M1 Mϕ, induced by signals such as interferon-gamma (IFN-γ) and lipopolysaccharide (LPS), display a potent antimicrobial activity, high production of pro-inflammatory cytokines (e.g., IL-6, TNF-α, IL-1β), and the ability to generate reactive oxygen and nitrogen species to eliminate pathogens (20, 21). These cells are essential in mounting effective immune responses but can also contribute to excessive inflammation and tissue damage when chronically activated (2). Conversely, M2 Mϕ, induced by cytokines like IL-4 and IL-13, are associated with tissue repair, fibrosis, and immunoregulation (22). They produce anti-inflammatory mediators such as IL-10 and TGF-β, promote extracellular matrix remodeling, and support angiogenesis (17). This phenotype is crucial in the resolution of inflammation after injury. However, sustained M2-like activation has been implicated in several pathological conditions, such as fibrosis (23, 24) and tumor prognosis (25–27), as they can enhance immune evasion and support tumor growth through angiogenic and immunosuppressive mechanisms (28). Although the classic dichotomy between pro- and anti-inflammatory macrophages is increasingly recognized as an oversimplification, particularly *in vivo* where macrophages exhibit a continuum of activation states, *ex vivo* models of polarized macrophage states remain valuable.

These reductionist systems, in which macrophages are differentiated with colony-stimulating factors (e.g., M-CSF or GM-CSF) and subsequently activated with defined stimuli such as IFN-γ (for M1) or IL-4 (for M2), offer controlled and reproducible conditions that facilitate the dissection of molecular mechanisms underlying macrophage differentiation, activation, and polarization (29).

Under these standardized conditions, when polarization is rigorously validated using multiple markers, the M1/M2 framework continues to be a widely accepted and practical tool within the immunology community (30). It enables meaningful comparisons across studies and laboratories, despite its limitations in fully capturing the dynamic and context-dependent nature of macrophage phenotypes *in vivo*.

The dynamic ability of Mϕ to transition between these states highlights their remarkable functional flexibility, which is strongly influenced by the surrounding microenvironment, as demonstrated by transplantation studies showing that, for example, peritoneal macrophages transplanted into the lung acquire an alveolar macrophage–like transcriptional profile, reflecting the dominant influence of local cues (31). Similarly, when embryonic TRMs are lost, for instance, due to irradiation or myocardial infarction, monocyte-derived macrophages can engraft in the pathological niches, adopt tissue-specific functions, and even achieve long-term self-maintenance through local proliferation (32, 33). This feature underscores the central role of Mϕ in maintaining a correct immune balance between either a pro- or anti-inflammatory behavior. Dysregulated Mϕ polarization indeed contributes to many pathological conditions, including chronic inflammatory diseases, cancer (34), and metabolic disorders (35). For instance, in atherosclerosis, prolonged M1-like activation contributes to plaque instability, while excessive M2 polarization may lead to unresolved inflammation and fibrosis (36). Similarly, in the tumor microenvironment, Mϕ often acquire an M2-like phenotype that facilitates tumor growth, immune evasion, and metastasis (37).

Importantly, the relative contribution of TRMs versus MDMs to disease pathogenesis can differ substantially. Embryonic-derived TRMs, due to their longevity and specialized adaptation to tissue niches, may drive chronic pathological remodeling when their homeostatic programs are dysregulated. For instance, Kupffer cell dysfunction contributes to steatohepatitis, alcoholic liver disease, intrahepatic cholestasis, activation or rejection of the liver during liver transplantation, and liver fibrosis (38, 39), while microglial hyperactivation promotes neurodegeneration (40). In contrast, MDMs are rapidly recruited during inflammation or injury, and are often central to fuel acute pathology by amplifying inflammatory cascades and tissue damage (41). Their persistence after resolution phases can also lead to maladaptive repair, such as fibrotic scarring in the lung of COVID-19 patients who had persistent respiratory symptoms (42, 43).

In the tumor microenvironment, MDMs often constitute the majority of tumor-associated macrophages (TAMs), where they are reprogrammed by tumor-derived signals into immunosuppressive, pro-tumoral phenotypes. TRMs, on the other hand, may contribute to tumor-promoting inflammation in some contexts, while in others, they retain protective or antitumor functions, depending on their origin, tissue context, and local cues.

Given their involvement in a wide range of physiological and pathological processes, Mϕ represent a critical focus of investigation for understanding their function and developing targeted therapeutic strategies.

However, the study of TRMs poses significant challenges, as various technical and biological limitations hinder their isolation and *ex vivo* study. A primary limitation is their relatively low abundance and anatomical localization within complex stromal and epithelial structures. This typically results in a low-yield of extraction, which is also labor-intensive and whose quality is too often user-dependent (44). Enzymatic digestion and mechanical dissociation, required for TRMs isolation, can alter their phenotype, leading to activation artifacts that may not accurately reflect their *in vivo* state (45, 46). Additionally, these cells rapidly lose their specialized characteristics when removed from their original environment, thus hindering efforts to maintain their functional identity *in vitro (*47*)*. Another major limitation is the availability of human tissues, which is restricted by ethical constraints, reliance on surgical resections, or *post-mortem* samples. Even when available, TRMs exhibit donor-to-donor variability due to differences in genetic background, environmental exposures, and underlying health conditions, which can influence their phenotype and function (48). All these factors hinder the reproducibility of experiments and significantly limit the ability to conduct large-scale studies.

Given these challenges, monocyte-derived macrophages (MDMs) have emerged as an accessible and widely used alternative for studying macrophage biology *in vitro*. MDMs can be obtained from peripheral blood monocytes, which are readily accessible from both healthy donors and patients through minimally invasive procedures (48–50). These cells can be differentiated into Mϕ under controlled culture conditions using well-established protocols involving growth factors such as macrophage colony-stimulating factor (M-CSF) (51). By exposing MDMs to specific cytokines or microbial components, it is possible to drive their polarization toward pro- (M1-like) or anti- (M2-like) inflammatory phenotypes, allowing the study of Mϕ activation, cytokine production, and functional responses (52).

MDMs represent indeed a highly versatile and physiologically relevant human-based model to investigate macrophage biology. They retain donor-specific immune characteristics, providing a faithful representation of primary human macrophage functional diversity. Their minimally invasive derivation from peripheral blood facilitates repeated sampling from the same donor, enabling longitudinal studies of immune responses and comparative analyses between healthy and diseased individuals. This accessibility, combined with the ability to polarize MDMs into distinct phenotypes (M0, M1, M2, or tissue-resident-like states), allows precise control over the experimental environment, including cytokine exposure, metabolic substrates, and co-culture with other cell types. Importantly, this flexibility supports high-throughput screening approaches for immunomodulatory compounds, biomarker discovery, and mechanistic studies of immune signaling pathways. By systematically integrating functional assays, transcriptomic profiling, and metabolic analyses, MDMs serve as a robust bridge between reductionist *in vitro* systems and the complexity of *in vivo* macrophage biology, offering a reproducible, scalable, and ethically accessible platform for both basic and translational research.

**Figure 1:**
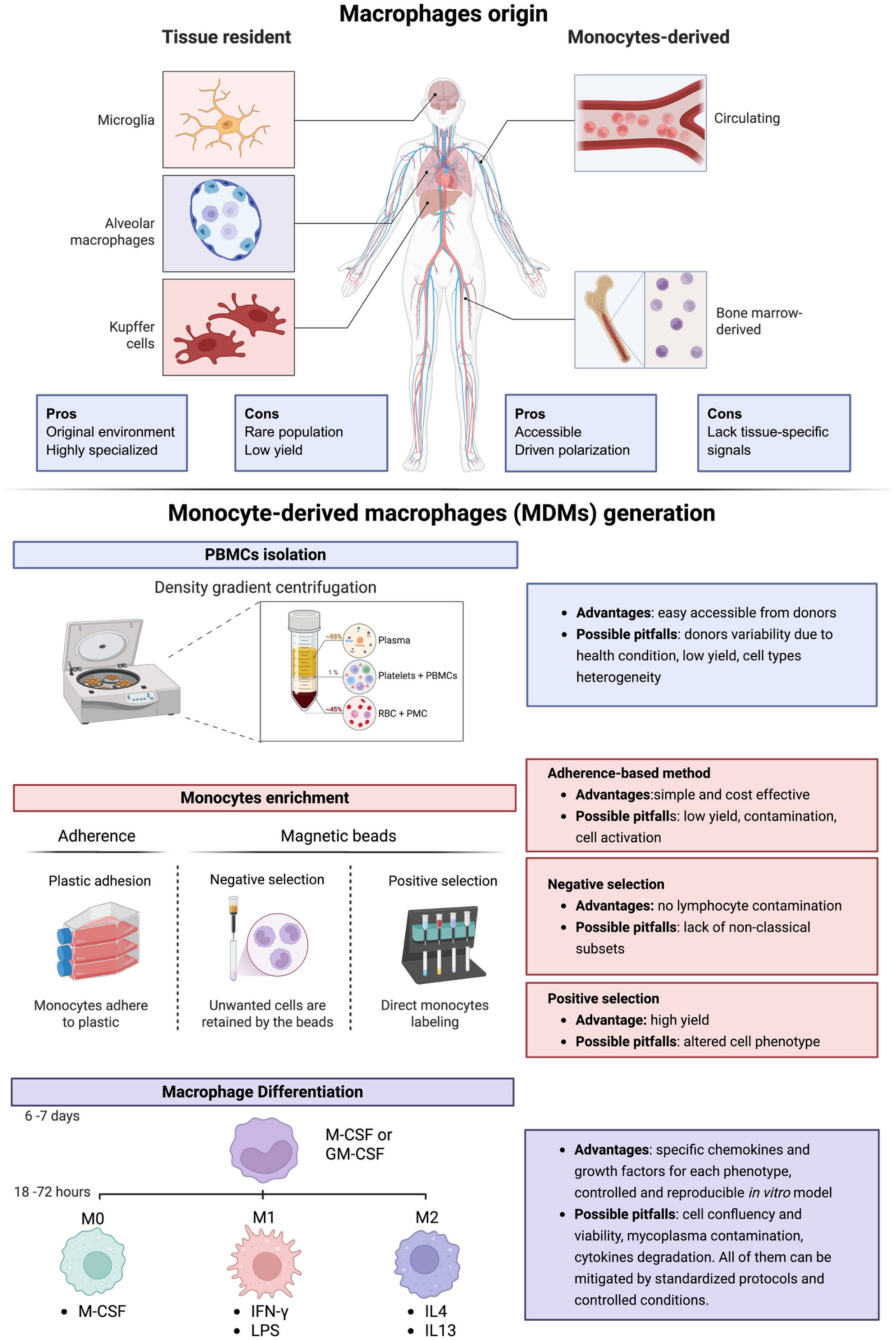
Macrophage origin and practical considerations for *in vitro* MDM generation. (Top) Schematic representation of macrophage ontogeny in humans, highlighting TRMs derived from embryonic progenitors, bone marrow–derived monocytes (BMDMs) and MDMs. Advantages and limitations of using TRMs, MDMs, and BMDMs in research applications are summarized. A schematic overview of human macrophage ontogeny, illustrating the origins of TRMs from embryonic progenitors, as well as bone marrow–derived monocytes (BMDMs) and MDMs, is presented. The main advantages and limitations of using these different macrophage sources in experimental settings are outlined to clearly understand biological relevance as well as technical feasibility. (Bottom) Step-by-step representation of the in vitro protocols for generating human MDMs. The process includes isolation of peripheral blood mononuclear cells (PBMCs), enrichment of monocytes through plastic adhesion, negative selection, or positive selection, followed by macrophage differentiation using specific cytokine cocktails and defined incubation times. For each step, practical pros and cons are provided.

However, despite their advantages, MDMs also have limitations, particularly in their ability to fully recapitulate the complexity and functional heterogeneity of TRMs. Unlike long-lived embryonically derived Mϕ, MDMs originate from circulating monocytes and lack the prolonged exposure to tissue-specific signals, which are critical to shape and maintain resident Mϕ identity. Consequently, they may not accurately model the transcriptional programs, epigenetic modifications, or functional specialization of macrophages in different organs. Nevertheless, MDMs remain a valuable, widely utilized model for investigating human Mϕ biology under highly controlled experimental conditions (**Figure 1**). Their differentiation *in vitro*, however, is still influenced by a range of technical variables, including culture conditions and the duration of differentiation, that can significantly impact cell yield, viability, and phenotype.

This review provides a comprehensive synopsis of the common human MDMs differentiation protocols, highlighting their advantages and technical pitfalls. We underline key factors that impact experimental reproducibility, discuss strategies to optimize macrophage culture conditions, and propose solutions to enhance the reliability of *in vitro* Mϕ models. By addressing these technical challenges, researchers can refine their methodologies, thereby improving the translational relevance of Mϕ-based studies.

While this manuscript focuses on human Mϕ, it is important to acknowledge that a substantial body of research relies on animal-derived macrophages (53) and *in vivo* animal models (54–58). Macrophage differentiation and function are known to vary significantly across species, reflecting differences in the immune system, tissue-specific cues, and developmental origins. For example, while murine models are widely employed for mechanistic studies, murine macrophages exhibit distinct transcriptional profiles, cytokine responses, and tissue-resident populations compared to their human counterparts, particularly in organs such as the liver, lung, and brain. Similarly, other species commonly used in research, including rats, non-human primates, and zebrafish, display unique macrophage biology that can influence experimental outcomes. These species-specific differences have critical implications when translating findings to human physiology or pathology. Therefore, while animal models provide indispensable insights into macrophage development, activation, and tissue homeostasis, careful consideration of interspecies variability is essential when interpreting results and extrapolating conclusions to human immunology. Integrating these insights with human-derived macrophage studies, including monocyte-derived and iPSC-derived models, can improve the physiological relevance of experimental findings and enhance translational potential.

## 2 Generation of M0, M1, M2 and TRMs-like MDMs

MDMs can be generated by isolating monocytes from human peripheral blood, typically obtained through blood donation. Monocytes are isolated from peripheral blood mononuclear cells (PBMCs) via density gradient centrifugation, a technique able separates blood components based on their density. In this method, diluted blood is carefully layered over a density gradient medium, such as Ficoll (59) or Percoll^®^, and then centrifuged to separate mononuclear cells based on their density (60, 61). The centrifugal force causes the separation of blood components into distinct layers: red blood cells and granulocytes, being denser, migrate to the bottom of the tube, while PBMCs, containing monocytes, locate at the plasma–medium interface. This PBMC layer can then be carefully harvested to isolate monocytes from the PBMC fraction using either adherence-based methods or magnetic bead-based immuno-isolation techniques (62). In adherence-based methods, PBMCs are plated onto tissue culture-treated plastic surfaces and typically incubated for 2 hours under standard culture conditions. Monocytes, which exhibit a natural tendency to adhere to plastic, attach firmly to the surface, whereas non-adherent cells, such as lymphocytes, can be removed by gentle washing (63). In contrast, magnetic bead-based immuno-isolation techniques offer higher specificity and purity. These methods rely on magnetic beads coated with antibodies against specific cell surface receptors. In negative selection protocols, antibody-conjugated beads bind to unwanted cells (*e.g.*, T cells, B cells, NK cells), which are then retained in a magnetic field, allowing untouched monocytes to be collected. Positive selection, on the other hand, involves directly labeling of monocytes using anti-CD14 antibodies, enabling their magnetic separation from the remaining PBMCs (63).

*Adherence-based method* is a simple and cost-effective technique that yields monocytes with a distribution of classical (CD14^++^CD16^−^ or CD14^+^CD16^−^), intermediate (CD14^++^CD16^+^ or CD14^+^CD16^+^), and non-classical (CD14^+^CD16^++^ or CD14^dim^CD16^+^) subpopulations, similarly to those present in PBMCs (64). However, this method has a significantly lower yield of monocytes, it is prone to contamination with lymphocytes and may induce pro-inflammatory activation due to the recognition of plastic as a non-physiological substrate, triggering receptor-mediated signaling and the release of pro-inflammatory cytokines (63, 65).

Negative selection avoids lymphocyte contamination but often results in extensive platelet contamination. Additionally, it selectively removes CD16-positive cells, producing a monocyte population dominated by classical monocytes, thereby lacking intermediate and non-classical subsets critical for specific functional studies.

Lastly, CD14-positive selection is effective in enriching monocytes, but alters cell phenotype, potentially due to the binding of anti-CD14 magnetic beads (66).

Thus, the choice of monocyte isolation method should be carefully tailored to the specific experimental aims to ensure the generation of a representative and functionally relevant monocyte population. The choice of isolation method directly influences downstream polarization efficiency – adhesion-based isolation preserves intermediate/non-classical monocyte subsets critical for M2 polarization, while CD14^+^ positive sorting enriches classical monocytes ideal for M1 induction.

After isolating CD14^+^ cells, monocytes can be differentiated into specific macrophage subtypes (M0, M1, M2) by exposing them to carefully designed cocktails of chemokines and growth factors. The cocktails are designed to mimic the microenvironmental factors that drive Mϕ polarization *in vivo*, thus providing a controlled platform to study the functional heterogeneity of Mϕ in a controlled *in vitro* setting. However, even within the differentiation of the same subtype, such as M1 or M2 Mϕ, there is significant variability in the protocols available.

Most commonly, monocytes are differentiated over 7 days with M-CSF (Macrophage Colony-Stimulating Factor) or GM-CSF (Granulocyte Macrophage-Colony Stimulating Factor) to produce naïve (M0) MDMs. M-CSF is constitutively present in serum and it is indispensable to drive TRMs and MDMs survival, proliferation and differentiation of myeloid progenitors into Mϕ (67–69). Under physiological conditions, M-CSF availability is tightly regulated through CSF-1 receptor (CSF-1R)-mediated endocytosis, establishing a feedback mechanism that regulates Mϕ proliferation according to the abundance of mature cells (70). Conversely, GM-CSF, found mostly at the sites of tissue inflammation, promotes a more activated Mϕ phenotype, characterized by strong antigen-presenting activity in response to pathogenic stimuli, and may also drive differentiation toward dendritic cells (DCs) (71). Accordingly, M-CSF- and GM-CSF-derived Mϕ exhibit different and unique transcriptional profiles (72–77).

Naïve (M0) macrophages can then be polarized into M1 and/or M2 Mϕ using IFN-y and lipopolysaccharide (LPS) (for M1) or IL-4 and IL-13 (for M2), among others, depending on the desired phenotype (*e.g.*, M2a, M2b, M2c). However, there is substantial variability in polarization protocols across studies, including differences in cytokine concentrations, timing of stimulation, and duration of exposure (**Table 1**). For instance, M0 MDMs can be differentiated from monocytes using M-CSF or GM-CSF, with cytokine concentrations ranging from 5 to 100 ng/ml and incubation periods of approximately 6–7 days (78–82), as shorter time spans (<5 days) often yield cells with incomplete maturation (83). Notably, high doses of M-CSF can impair myeloid differentiation, promoting a shift toward a dendritic cell-like phenotype (84). Additionally, M-CSF stimulation can drive morphological differentiation into larger, macrophage-like cells with altered inflammatory potential (85). In general, MDMs induced by these factors display distinct phenotypes and functions: cells induced by GM-CSF actively participate in antigen presentation and produce inflammatory mediators, such as IL-12, IL-23 and tumor necrosis factor (TNF)-α (86), while M-CSF-induced cells are mostly involved in scavenging and phagocytosis and release anti-inflammatory cytokine IL-10 (87).

**Table 1:** Examples of published protocols for the *in vitro* differentiation and polarization of human MDMs.

M0			M1			M2				
M-CSF (ng/mL)	GM-CSF (ng/mL)	Time (days)	INF-γ (ng/mL)	LPS (ng/mL)	Time (hours)	IL-4 (ng/mL)	IL-13 (ng/mL)	IL-10 (ng/mL)	Time (hours)	Ref
	10	7								(49)
20	25	6	20	100	72	20			72	(50)
100		7		100	18	20			18	(51)
100	25	7								(52)
100		7								(53)
50		7	25	5	48			20	48	(54)
100		7	25	50	72	25			72	(55)
			100	100	24 (INF-γ) 3 (LPS)	10	10		24	(56)
100		6	2	50	48	10	10		48	(57)
5		6	100			10				(61)
50		6	20	20	48	20	20		48	(73)
40		6	20	20	24	20			24	(82)

The table depicts representative protocols used to generate non-polarized (M0), classically activated/ pro-inflammatory (M1), and alternatively activated/ anti-inflammatory (M2) macrophages from human peripheral blood monocytes. Key reported parameters include the type of cytokines used, the concentrations, and the stimulation period.

The M0 cells can then be further polarized into either M1 or M2 phenotypes. M1 Mϕ are typically generated by stimulating M0 cells with a combination of IFN-γ (20–100 ng/ml) and LPS (5–100 ng/ml) for 18 to 72 hours (13, 88). In contrast, M2 polarization is most commonly achieved by exposing M0 Mϕ to T-helper 2 (Th2) cytokines, i.e., IL-4 (10–20 ng/ml), IL-13 (10–20 ng/ml), or IL-10 (20 ng/ml) for similar durations (18–72 hours) (88–91). Different conditions critically influence Mϕ activation status, the phenotypic marker expression, and, more importantly, functional outcomes, highlighting the necessity for careful selection of differentiation conditions to achieve reproducible and physiologically relevant Mϕ phenotypes (**Figure 1**). Among surface markers associated with M1 polarization, CD64 (FcγRI) and CD80 are commonly reported. However their expression is influenced by the type and duration of the polarizing stimuli. IFN-γ stimulation induces a robust upregulation of CD64, consistent with its role as a high-affinity Fc receptor that mediates enhanced phagocytosis and antibody-dependent functions in classically activated macrophages. In contrast, CD80, a costimulatory molecule involved in T-cell activation, is most prominently induced following exposure to LPS, either alone or in combination with IFN-γ (92). CD40, another costimulatory molecule, exhibits relatively stable expression across different polarization protocols (86). CD163, a hemoglobin–haptoglobin scavenger receptor frequently used as a marker of alternatively activated macrophages, shows variable expression depending on the cytokine used. Notably, IL-4 alone is insufficient to strongly induce CD163 in M0 macrophages, indicating that not all M2-polarizing stimuli uniformly drive its expression (86).

Importantly, extending the duration of cytokine stimulation does not necessarily promote further maturation, as prolonged stimulation can alter cells responsiveness. For example, LPS-induced M1 macrophages exhibit peak expression of CD86 and HLA-DR at 12 hours post-stimulation, followed by a decrease at 24–72h (93), possibly due to the production IL-10 that which inhibits CD86 in an autocrine manner to prevent overexpression (94). In the same cells, CD64 expression gradually increased between 8 and 72 hours, whereas HLA-DR expression peaked at 12 hours (95). Regarding M2 MDMs, the expression of the surface markers CD200R and CD206 gradually increased over time in human MDMs polarized using IL-4, reaching the maximum expression level between 48 and 72 h (95). The surface expression of the scavenger receptor CD163 was increased after 24–72 h of IL-10 stimulation (86, 93, 96*).*

These findings underscore the importance of both stimulus composition and timing in shaping macrophage surface marker expression and highlight the complexity of interpreting macrophage phenotypes across experimental systems.

Interestingly, Mϕ differentiation can also occur in the absence of exogenous cytokine stimulation, a process known as spontaneous monocyte-to-Mϕ transition (*i.e.*, in the absence of specific cytokine stimulation) (97, 98). In this case, monocytes are typically cultured in serum-containing media. Within 7 days, a clear differentiation in terms of cytoskeleton architecture, lipid content, and macropinocytosis/efferocytosis capacity was observed compared to undifferentiated monocytes. Flow cytometry analysis revealed a predominance of the M2 over the M1 phenotype. Notably, the expression of M1 and M2 markers was not mutually exclusive, indicating a mixed polarization state within the resulting population (98). This evidence further confirms the phenotypic complexity of *in vitro* macrophage models and the importance of rigorously defining differentiation and polarization protocol conditions.

Importantly, MDMs lack long-term exposure to the complex microenvironmental cues present in the tissues that shape tissue-resident Mϕ identity *in vivo*. These cues include cytokine gradients, cellular interactions, and metabolic conditions specific to tissues such as the lung, liver, or gut (99). As a result, MDMs often fail to fully recapitulate the phenotypic and functional heterogeneity observed among TRM subsets across different tissues. To address these limitations, differentiation protocols incorporating specific cytokines and growth factors have been developed to guide MDMs toward specialized phenotypes that more closely resemble TRMs or macrophages found in particular pathological contexts. For instance, alveolar-like Mϕ, which are critical for lung homeostasis and host defense, can be generated using a combination of GM-CSF, TGF-β, and IL-10, supplemented with natural bovine surfactant. This approach mimics the pulmonary microenvironment, where GM-CSF drives alveolar Mϕ differentiation, TGF-β supports tissue adaptation, and IL-10 enhances their immunoregulatory properties. The resulting cells demonstrate hallmark features of resident alveolar macrophages, including high phagocytic capacity, adaptations in lipid metabolism, and a balanced inflammatory response. These properties make them particularly valuable for modelling respiratory infections, pulmonary inflammation, and lung-specific immune responses (100).

Similarly, tumor-associated macrophage (TAM)-like phenotypes can be induced by exposing MDMs to a cytokine cocktail containing IL-4, IL-10, M-CSF, and tumor-conditioned media, an approach that simulates the immunosuppressive tumor microenvironment. IL-4 and M-CSF promote an M2-like phenotype characterized by tissue remodeling and immunosuppression, while IL-10 further enhances their anti-inflammatory properties. The inclusion of tumor-conditioned media further skews Mϕ toward a pro-tumoral phenotype, resembling TAMs in the tumor microenvironment. These cells typically exhibit high expression of typical anti-inflammatory markers (*i.e.*, CD163 and CD206), increased secretion of immunosuppressive cytokines like TGF-β, and enhanced angiogenic and matrix-remodeling abilities. TAM-like MDMs are particularly useful for studying tumor-immune interactions, investigating immune evasion mechanisms, and exploring therapeutic strategies targeting the tumor microenvironment (101–106). However, despite these advances, it remains debated how faithfully MDM-derived models can reliably reproduce TRM identities. Recent work by Elchaninov et al. demonstrated that attempts to replicate the hepatic niche using liver-conditioned media failed to induce MDMs to fully adopt the phenotype of Kupffer cells—the resident macrophages of the liver. This highlights the critical role of ontogeny and long-term niche-specific imprinting in shaping TRM identity, which cannot be fully replicated through cytokine supplementation alone (107).

Together, these findings underscore both the versatility and limitations of MDM-based models. While cytokine-guided differentiation offers a powerful approach to approximate specific macrophage phenotypes, especially for disease modeling and therapeutic screening, such models fall short of recapitulating the full complexity of TRMs shaped by developmental origin and sustained tissue residency. Moving forward, integrating MDM systems with advanced tissue-engineered platforms, organoids, and multi-cellular co-culture models may offer more physiologically relevant systems that bridge the gap between *in vitro* plasticity and *in vivo* fidelity.

Possible pitfalls when differentiating cells:

- Confluency: One critical factor that influences Mϕ differentiation is cell confluency. The density at which monocytes are plated plays a key role in determining their viability, differentiation efficiency, and polarization outcomes. Macrophages strongly rely on cell-to-cell interaction and paracrine signals. Under low-density conditions (*i.e.*, below 3x10^5^ cells/ml), monocytes may remain undifferentiated or even undergo apoptosis instead of successfully differentiating. On the other hand, high-density cultures (> 1x10^6^ cells/ml) can result in Mϕ fusion and multinucleated giant cell formation, particularly when M-CSF is used, or when the cells are kept in culture for a long time. Importantly, while low-density culture can lead to unwanted differentiation toward an M1 profile, over-confluency can instead promote an M2-like polarization bias, as cell-to-cell contact increases IL-10 and TGF-β secretion. However, overcrowded cultures can lead to nutrient depletion and waste accumulation over time, impairing macrophage functionality and inducing stress responses. The majority of the published studies use a cell density between 0.5×10^6^ to 1×10^6^ cells/ml (79, 80, 108, 109), as this range optimally balances differentiation efficiency, viability, and functional polarization while minimizing apoptosis at lower densities and excessive fusion at higher densities.

- Mycoplasma contamination: Mycoplasma contamination is usually “asymptomatic”, meaning it does not usually cause cell death, but it can cause several alterations in cultured cells (110). These effects include an altered metabolism, reduced growth rates, and chromosomal aberrations. Even though not immediately noticeable, these changes can significantly impact the reliability of the experiments. Notably, mycoplasma contamination has been shown to trigger the release of pro-inflammatory cytokines, such as IL-1β, IL-6, and TNF-α, from human PBMCs, including monocytes (111). In preliminary experiments, M1-polarized MDMs unexpectedly upregulated IL-10 during LPS/IFN-γ stimulation, which deviates from the typical pro-inflammatory profile. PCR screening revealed low-level mycoplasma contamination as the cause. After introducing weekly mycoplasma testing and filter-sterilizing all media and cytokine stocks, IL-10 levels returned to baseline and CD86 expression increased by approximately 40%, restoring a canonical M1 phenotype. This example illustrates how undetected contamination can profoundly alter macrophage polarization, and highlight the importance of strict quality control measures in MDM culture.

Therefore, it is strongly recommended to regularly screen for mycoplasma contamination using a PCR or enzymatic detection kit.

- Cytokines degradation: The differentiation of MDMs strongly relies on the use of pro- and anti-inflammatory cytokines, which must remain stable and biologically active throughout the differentiation process. However, cytokine integrity can be easily compromised by degradation (*i.e.*, M-CSF and GM-CSF), improper storage, repeated freeze-thaw cycles (*i.e.*, IL-4, IL-13, and IFN-γ), and prolonged incubation at suboptimal temperatures, all of which may lead to inconsistent differentiation outcomes and experimental variability (112). To preserve cytokine activity and ensure consistent MDMs differentiation, cytokines should be stored in single-use aliquots at 2-8 °C up to four weeks, at -20 °C for up to 6 months, or -80 °C for long-term storage, to prevent repeated freeze-thaw cycles. In general, most cytokines are stable for up to three freeze–thaw cycles (113). These proteins should be reconstituted in a stabilizing buffer (*e.g.*, 0.1% BSA in PBS) to enhance stability, and the prolonged incubation time at room temperature should be avoided. Moreover, the culture media should be replenished every 2/3 days with freshly resuspended cytokines for long differentiation protocols, to maintain a stable cytokine concentration (99).

- Altered cells’ viability: Cell viability can vary throughout the differentiation process, particularly in the M1 MDMs phenotype. Several factors influence cell viability, including the choice of culture medium. Notably, the use of Dulbecco’s Modified Eagle Medium (DMEM) instead of Roswell Park Memorial Institute 1640 Medium (RPMI-1640) has been reported to impact MDMs survival and function (114). In general, to ensure results reliability, it is essential to monitor cell viability throughout the study. Various assays are available for this purpose, including the Trypan blue exclusion, MTT or ATP assays, and live/dead staining for flow cytometry. However, it is important to consider potential biases in viability assessments. For instance, live/dead staining may underestimate cell death, as detached dead cells are often lost during medium removal. Selecting an appropriate method and complementing it with additional viability assays can help ensure accurate assessment of cell health, increasing experimental reproducibility.

- Low MDMs yield: There are several reasons behind the low MDMs yield. For example, cells might not correctly adhere to the surface, or the monocytes might not be fully separated, leading to high contamination from other cell types (such as granulocytes and lymphocytes). To improve monocyte isolation, the selection of separation methods is crucial. Using density gradient centrifugation (*e.g.*, Ficoll-Paque), followed by magnetic-activated or fluorescence-activated cell sorting, can help increase purity. Adherence-based selection should be optimized by testing plating conditions, including the type of culture dish and incubation time.

The choice of serum during monocyte-to-macrophage differentiation is a critical factor influencing both yield and functionality. Fetal Bovine Serum (FBS) alone can significantly reduce the yield of monocyte-derived macrophages compared to autologous serum (115). This reduction is likely due to the absence of key growth factors and cytokines in FBS that are naturally present in autologous serum, which better support monocyte differentiation, improving both yield and functionality. If autologous serum is not available, heat-inactivated human AB serum serves as a viable alternative.

Donor’s health condition also plays a role in differentiation efficiency. Monocytes isolated from individuals undergoing treatment with immunosuppressive agents, corticosteroids, or other drugs affecting the immune system, may exhibit a compromised differentiation capacity. In such cases, selecting healthy donors or carefully evaluating medication history is essential to ensure optimal differentiation outcomes.

## 3 Phenotypic characterization of M0, M1, M2 MDM

Prior to polarization into M1 or M2 phenotypes, monocytes first differentiated into M0 MDMs. These cells exhibit a baseline, inactivated state, characterized by a minimal inflammatory or immunomodulatory activity. M0 Mϕ typically possess a more homogeneous and round morphology, with a relatively smooth surface and a less pronounced cytoskeletal organization than their activated counterparts. Upon differentiating into M1 or M2 MDMs, cells change their morphology in accordance with their functional roles. M1 cells undergo a strong cytoskeletal reorganization that leads to a more pronounced surface area, showing an elongated or irregular shape and filopodia (116). In contrast, M2 Mϕ exhibit a more rounded and spindle-shaped appearance, with giant multinucleated cells, a smoother cytoplasmic outline, and a less dynamic actin filament network than M1 (117).

**Figure 2:**
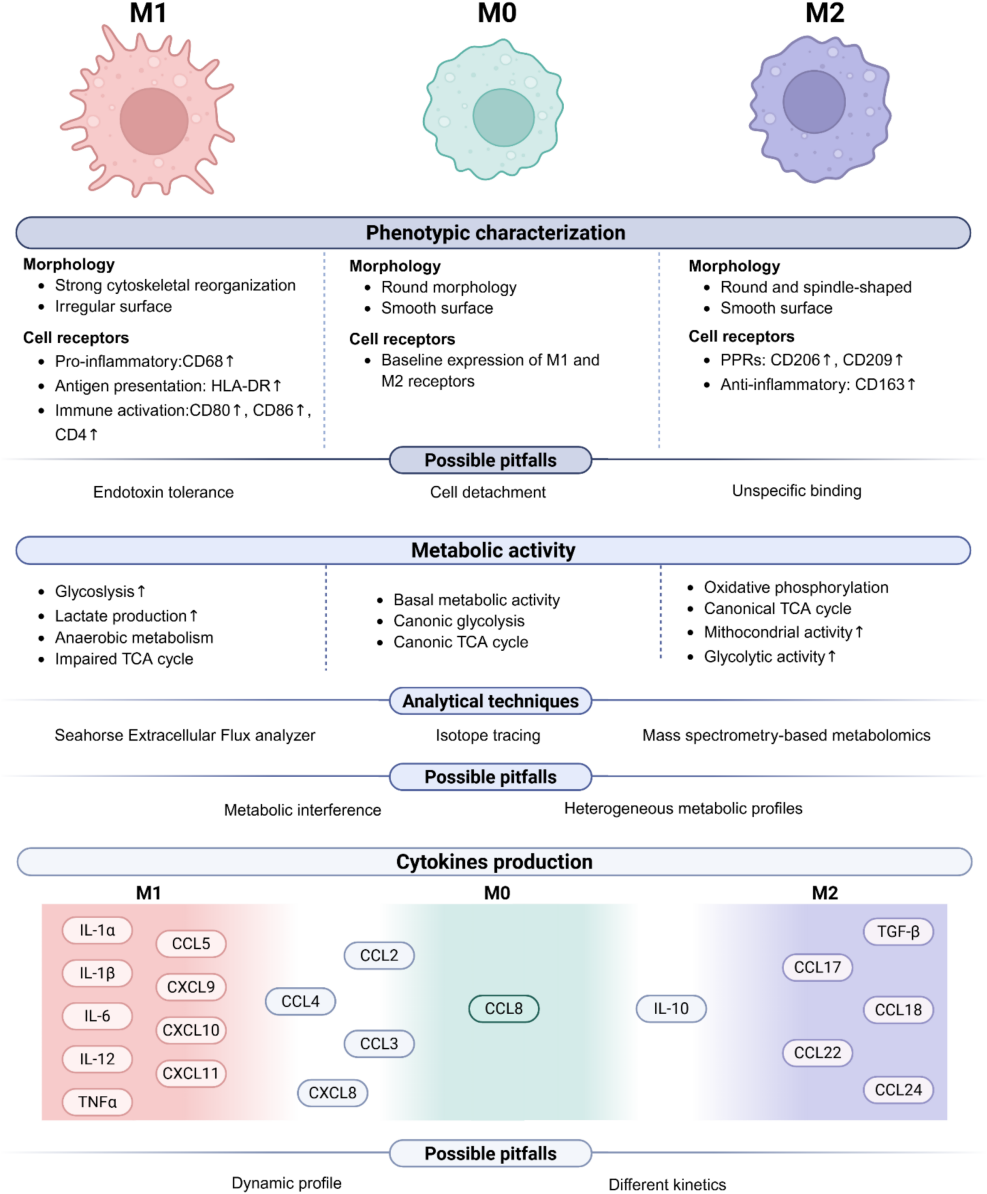
Phenotypic, metabolic, and functional characterization of M0, M1, and M2 MDMs. (Top) Overview of phenotypic features used to distinguish M0, M1-like, and M2-like MDMs, including differences in cell morphology and surface receptor expression. Possible experimental pitfalls are listed. (Middle) Summary of metabolic rewiring associated with each macrophage phenotype. Techniques commonly used to assess these metabolic changes are shown, along with their respective possible limitations. (Bottom) Cytokine profiles typically associated with each MDM's phenotype are presented. Methodological pitfalls are also indicated to guide the accurate interpretation of functional data.

In addition to morphological changes, MDMs differentiation is marked by extensive changes in cell receptor expression patterns, indicative of their polarization into specific functional phenotypes. Recent technological advances in high-dimensional flow cytometry have revolutionized macrophage immunophenotyping. Contemporary cytometers, capable of simultaneously detecting 30–40 fluorochromes, now allow for precise discrimination among Mϕ subsets with unprecedented precision, enabling detailed correlation between receptor expression pattern and functional polarization states (118). M1 cells exhibit a robust pro-inflammatory phenotype characterized by elevated expression of key surface markers involved in antigen presentation and immune activation (17, 52). These include CD68, a lysosomal glycoprotein associated with phagocytic activity; HLA-DR, the major histocompatibility complex class II (MHC-II) molecule essential for antigen presentation to CD4+ T cells; and co-stimulatory molecules such as CD86, CD80, and CD40, which are critical for T-cell activation. Additionally, M1 Mϕ upregulate CD64 (Fc gamma receptor I), enhancing their ability for antibody-dependent phagocytosis, and TLR2, a pattern recognition receptor involved in detecting microbial components and initiating inflammatory signaling cascades. Conversely, M2 Mϕ, which are associated with anti-inflammatory and immunomodulatory functions, express a completely different repertoire of surface markers (17, 52). Among these molecules, there are pattern recognition receptors (PRRs) like CD206 (mannose receptor) and CD209 (DC-SIGN). These PRRs recognize carbohydrate structures present in pathogens, including fungi, bacteria, and viruses, and facilitate their phagocytosis and internalization. Another hallmark of M2 cells is the class I scavenger receptor CD163, which participates in the removal of apoptotic cells, thus contributing to the resolution of inflammation and the promotion of tissue repair (**Figure 2**).

While the M1/M2 classification provides a useful framework for understanding Mϕ functional state, it should not be considered a rigid binary categorization. Mϕ phenotype exhibits significant heterogeneity *in vivo*, reflecting a high degree of plasticity and adaptability in response to different environmental stimuli. This functional flexibility suggests that M1- and M2-associated markers are not mutually exclusive (1). Mϕ express different levels of these markers based on the combination of signals they encounter. The key distinction between M1 and M2 is thus defined more accurately by the relative expression levels of phenotype-associated markers, rather than either absolute presence or absence. This differential expression enables cells to finely tune their functional responses according to the different tissue-specific demands and pathological conditions.

*Ex vivo*, MDMs stimulated with M-CSF are referred to as non-polarized or naïve Mϕ (M0). While these cells possess basic phagocytic activity and the ability to detect pathogens, they require further polarization signals to acquire the full spectrum of specialized macrophage functions, whether pro-(M1) or anti- (M2) inflammatory (119). Phenotypically, M0 Mϕ can be characterized by a balanced expression of both M1- and M2-associated surface markers, with neither subset dominating. Such an intermediate phenotype represents an intrinsic Mϕ baseline readiness to shift toward either M1 or M2 polarization state upon specific stimulation (**Figure 1**).

Importantly, this plasticity is not limited to the M0 state. The phenotypic identity of polarized MDMs (M1 or M2) is also dynamic and can revert over time. It has been reported that following six days in cytokine-free medium, M1 and M2 Mϕ progressively lose their polarized profiles, further highlighting the reversible nature of Mϕ activation and the central role of environmental cues in shaping their functional state (120).

Possible Pitfalls:

- Endotoxin Tolerance (ET): During *in vitro* differentiation of Mϕ, particularly toward the M1 phenotype, it is critical to account for the phenomenon of endotoxin tolerance. This refers to an altered state of responsiveness to secondary stimulation with LPS, in which Mϕ exhibit a diminished or altered inflammatory response (121). Mϕ undergoing endotoxin tolerance display an increased phagocytic activity, marked by overexpression of CD64, coupled with a conserved capacity to kill internalized pathogens. However, this comes together with an impaired antigen presentation function, as evidenced by reduced levels of MHC-II and CD86. This acquired phenotype is not transient, as endotoxin tolerance is long-lasting, with persistent alterations in Mϕ behavior. In support of this, decreased HLA-DR expression in endotoxin-tolerant cells has been related to impaired T-cell proliferation as well as reduced production of IFNϒ by T-lymphocytes (122).

From a physiological perspective, ET has important implications for understanding immune responses in conditions such as sepsis. The reduced pro-inflammatory ability of endotoxin-tolerant macrophages, coupled with the upregulation of anti-inflammatory cytokines, contributes to limiting systemic inflammation and thus mitigating the risk of septic shock. At the same time, the enhanced phagocytic activity facilitates effective bacterial clearance, which is crucial for resolving infections. However, the impaired antigen presentation in endotoxin-tolerant macrophages suggests that the development of a robust adaptive immune response is hindered, avoiding excessive immune reactions (121).

ET in macrophages is typically induced by prolonged or high-dose exposure to LPS. Robust tolerance generally requires 16–48 hours of low-dose LPS stimulation, with higher concentrations, ranging from 10 ng/mL to 1 μg/mL, producing a stronger tolerogenic phenotype (123). Although the kinetics of ET have not been systematically characterized across all macrophage models, *in vitro* studies suggest that key features, including increased phagocytic capacity and impaired antigen presentation, can persist for up to five days following LPS exposure (124).

These observations highlight the importance of carefully controlling both LPS dose and exposure duration in experimental designs, particularly when differentiating or polarizing macrophages, to avoid inadvertent induction of ET and its confounding effects on downstream assays.

If tolerant macrophages are used, for example, in experiments to assess inflammatory activation, pathogen response, or T-cell stimulation, they may produce misleading results. In fact, a diminished cytokine response could be erroneously interpreted as a weak stimulus effect, impaired macrophage function, or experimental failure, rather than a pre-existing tolerant state. Similarly, impaired antigen presentation may lead to false conclusions about adaptive immune activation, while skewed functional readouts can generate contradictory or non-reproducible data. Therefore, rigorous control of LPS exposure, both in concentration and timing, is essential during macrophage differentiation, stimulation, or polarization. Equally important is the evaluation of activation state prior to downstream functional assays to ensure the accurate attribution of observed responses to the intended experimental conditions.

Different strategies can be used to avoid the induction of endotoxin tolerance during monocyte differentiation into Mϕ. One of the most straightforward approaches involves precise control over the concentration (<10 ng/mL) and duration (24h) of the LPS exposure during differentiation. Using low concentrations of LPS or limiting the exposure time can reduce the risk of inducing endotoxin tolerance phenotype, while still effectively differentiating macrophages into the M1 subset. The absence of endotoxin tolerance can be easily assessed by subjecting M1 Mϕ to a secondary LPS challenge. Cells that are endotoxin-tolerant will exhibit attenuated cytokine production (especially TNF-α and IL-6) and downregulation of MHC-II and costimulatory molecules upon re-exposure to LPS (122).

These insights emphasize the necessity for meticulous standardization in macrophage-based *in vitro* models. The dynamic responsiveness of macrophages to environmental cues such as LPS underscores their functional plasticity but also reveals their vulnerability to unintended conditioning. Without strict experimental oversight, phenomena like endotoxin tolerance can silently reshape macrophage behavior, compromising data integrity and hindering the reproducibility of findings. Recognizing and accounting for these variables is essential for advancing macrophage-focused research with both mechanistic depth and translational relevance.

-Cell detachment: Upon differentiation, Mϕ adhere firmly to the culture plates, making cell detachment challenging. Therefore, selecting an appropriate detachment method is critical to preserve cell viability and integrity for the following experimental applications, such as flow cytometry. While trypsin is commonly used, it cleaves peptides after lysine or arginine residues, and it can degrade a wide range of surface proteins, depending on incubation time, potentially altering the phenotype of cells (125, 126). As a milder enzymatic cell detachment buffer, Accutase is recommended for detaching adherent Mϕ, as it minimizes enzymatic damage while preserving surface marker expression (127). EDTA chelates calcium ions required for integrin-mediated adhesion and allows for mild detachment with minimal impact on surface proteins. However, for strongly adherent cells, ethylenediaminetetraacetic acid (EDTA) alone is often insufficient and must be combined with mechanical dislodgement by scraping, which may inadvertently damage cells. Mechanical detachment with a cell scraper, while enzyme-free, also requires careful handling to minimize physical stress. Thus, the choice of detachment strategy must balance the need for cell recovery with the preservation of specific surface markers and cell viability.

-Unspecific binding of antibodies to FcY receptors: Mϕ express high levels of Fcγ receptors (FcγRs), including FcγRI (CD64), FcγRII (CD32), and FcγRIII (CD16), which can bind the Fc portion of antibodies in an antigen-independent manner. This non-specific binding is particularly problematic in immunophenotyping assays, such as flow cytometry and immunostaining, as it can lead to false-positive signals and consequent misinterpretation of surface marker expression (128). This problem is particularly relevant when using mouse monoclonal antibodies against human Mϕ markers, as these antibodies strongly interact with Fcγ receptors, independently of antigen specificity. Therefore, it is fundamental to block Fcγ receptors prior to antibody staining. This can be easily achieved by pre-incubating Mϕ with human IgG or commercially available FcR-blocking reagents to saturate these receptors and minimize non-specific interactions. Additionally, using F(ab’)2 antibody fragments, which lack the Fc region, can further reduce FcγR-mediated binding. Appropriate experimental controls, such as isotype controls, should always be included to distinguish specific from non-specific staining. Also, titration of antibody concentrations should be carefully tested to avoid artifacts arising from receptor saturation.

## 4 Metabolic activity of differentiated MDM

Recently, metabolic studies have revealed how polarization of MDMs is linked to the metabolic reprogramming of the cells, with distinct metabolic profiles characterizing classically and alternatively activated MDMs (**Figure 2**). M1 cells predominantly rely on glycolysis to support their heightened pro-inflammatory function by rapidly generating ATP and biosynthetic precursors that are essential for cytokine production and antimicrobial activity. This metabolic shift is reflected in their increased glycolytic ability, elevated lactate production, and reduced oxygen consumption rate (OCR), collectively indicating a preference toward anaerobic metabolism. Furthermore, M1 MDMs exhibit an impaired tricarboxylic acid (TCA) cycle, with the accumulation of key intermediates such as citrate (required to produce nitric oxide and prostaglandins) and succinate (fundamental to promote the expression of pro-inflammatory cytokines, such as IL-1β) (129). This altered TCA cycle supports the inflammatory phenotype of M1 MDMs by increasing the production of immune effector molecules at the expense of oxidative phosphorylation.

Conversely, M2-MDMs rely predominantly on oxidative phosphorylation (OXPHOS) and fatty acid oxidation to sustain their anti-inflammatory and tissue-repairing functions (130). Unlike M1 Mϕ, M2 cells maintain a canonical TCA cycle and display enhanced mitochondrial respiratory ability, enabling efficient ATP production under steady-state conditions. Interestingly, M2-MDMs also display elevated glycolytic activity relative to unpolarized M0 macrophages, indicating a metabolic flexibility that may facilitate adaptive responses under stress conditions (131).

Metabolic modulation during MDM polarization can significantly alter the phenotypic and functional outcomes of these cells. For example, inhibition of glycolysis using 2-deoxy-d-glucose (2-DG) or dichloroacetate (DCA) during M2 polarization has been shown to alter their metabolic balance, leading to a shift in their cytokine production profile (132). Specifically, glycolysis inhibition reduces IL-10 secretion, a key anti-inflammatory cytokine associated with M2 function; while simultaneously increasing the production of IL-6, a cytokine commonly linked to inflammatory responses. Moreover, glycolysis inhibition affects the transcriptional landscape of M2 MDMs, altering the expression of key genes involved in their function. This includes changes in the colony-stimulating factor 1 (CSF1), which regulates Mϕ survival and differentiation, in the matrix metalloproteinase-9 (MMP9), involved in extracellular matrix remodeling, and in the vascular endothelial growth factor-A (VEGF-A), a key mediator of angiogenesis. These alterations suggest that metabolic shifts can influence not only cytokine production but also tissue remodeling and pro-angiogenic functions of M2 cells. Despite these strong metabolic changes, M2 MDMs differentiated in the presence of glycolysis inhibitors retain a degree of functional plasticity. Upon exposure to appropriate stimuli, they can still produce IL-10, underscoring the dynamic and adaptable nature of macrophage metabolism (132).

A range of analytical techniques can be employed to analyze MDMs metabolism, thus providing insights on how metabolic shifts influence Mϕ function. Among the most widely used platforms is the Seahorse Extracellular Flux (XF) analyzer, which enables real-time, label-free measurement of cellular bioenergetics (133). It evaluates metabolic alterations through the Cell Mito Stress Test, which simultaneously quantifies extracellular acidification rate (ECAR), indicative of glycolysis, together with oxygen consumption rate (OCR) as a marker of oxidative phosphorylation (OXPHOS). The assay employs metabolic modulators such as oligomycin (ATP synthase inhibitor), FCCP (to assess maximal mitochondrial respiration), and a combination of rotenone and antimycin A (to shut down mitochondrial respiration and measure non-mitochondrial oxygen consumption) (134–136).

Beyond Seahorse XF analysis, additional approaches have been developed to further characterize MDM metabolic dynamics (**Figure 2**). Stable isotope tracing using C-13 labelled glucose, glutamine, or fatty acids allows tracking of the metabolic fate of key substrates, providing deeper insights into TCA cycle activity, amino acid metabolism, and lipid catabolism. Fluorescence-based metabolic sensors, such as genetically encoded biosensors for ATP, NAD^+^/NADH, and lactate, offer real-time imaging of metabolic dynamics at the single-cell level. Mass spectrometry-based metabolomics provides a comprehensive profiling of metabolic intermediates, allowing for the identification of distinct MDMs metabolic signatures in response to different stimuli. Additionally, flow cytometry-based metabolic assays, including MitoTracker staining for mitochondrial mass and membrane potential, can be integrated with polarization markers to define metabolic heterogeneity within MDM populations. Together, these complementary approaches provide a multidimensional understanding of MDM metabolism, enabling researchers to obtain a clearer overview of MDM metabolic dynamics.

Possible pitfalls.

- Metabolic interference: Cell viability is a critical factor influencing metabolic assessments. Endotoxin contamination and the use of metabolic inhibitors, such as 2-deoxy-D-glucose (2-DG) and dichloroacetate (DCA), can induce cellular stress or apoptosis, potentially confounding data interpretation. This may significantly impact metabolic measurements, particularly those involving mitochondrial respiration or glycolytic flux. To minimize such contribution, cell viability should be systematically monitored using established methods, such as trypan blue exclusion, ATP-based viability assays, or flow cytometry-based approaches. To further characterize the type of cell death, staining with Annexin V/7-AAD or caspase activity assays can differentiate between apoptosis and necrosis, both of which may significantly alter metabolic readouts.
- Heterogeneous metabolic profiles due to incomplete polarization: The differentiation and polarization of MDMs must be tightly controlled, as incomplete or heterogeneous polarization can lead to mixed metabolic profiles within the same experimental group. This variability significantly impacts the reproducibility and data interpretation. To mitigate this issue, a well-established differentiation protocol should be employed, incorporating defined cytokine exposure times, serum sources, and medium compositions. Functional validation of M1/M2 polarization using surface markers (*e.g.*, CD80/CD86 for M1, CD206/CD163 for M2) and cytokine profiling is essential before performing metabolic studies.

Technical Considerations Associated with the Seahorse XF Method for MDMs.

- Impact of cell detachment on metabolic state: MDMs are adherent cells, and their detachment, whether via enzymatic digestion or non-enzymatic dissociation buffers, can rapidly induce metabolic alterations. This process may transiently change mitochondrial respiration and glycolysis, potentially altering metabolic assessments. Furthermore, residual detachment reagents can interfere with the Seahorse assay buffer components, further compromising data accuracy.
- Cell reattachment and distribution in Seahorse XF plates: Following transfer, some MDMs may exhibit suboptimal adhesion to the Seahorse XF plate, resulting in uneven cell distribution, aggregation, or cell loss. Such inconsistencies can introduce substantial well-to-well variability in OCR and extracellular acidification rate ECAR, thereby compromising assay reproducibility. To minimize this issue, the Seahorse XF plate should be pre-coated with an appropriate substrate (*e.g.*, poly-D-lysine, fibronectin, or collagen) to enhance cell attachment.

Optimization of cell density is also critical, as excessive confluency may cause localized oxygen depletion before the assay begins, leading to artificially low OCR values. Conversely, insufficient cell density may result in unreliable metabolic measurements due to inadequate signal detection.

Normalizing metabolic readouts to cell number, obtained through direct counting, reduces technical variability and ensures that observed metabolic changes reflect biological differences rather than inconsistencies in sample preparation (137).

- Environmental adaptation to Seahorse XF plates and metabolic reprogramming: Transitioning MDMs from standard culture plates to Seahorse XF plates may induce metabolic adaptation that can transiently alter their metabolic state. The change in surface (plastic *vs* Seahorse plate) can indeed influence cell adhesion and morphology. Moreover, the seahorse assays require unbuffered media (*e.g.*, Seahorse XF DMEM or RPMI), which differs from standard culture media and can transiently alter metabolic activity. Additionally, oxygen availability differs between standard culture plates and Seahorse XF plates. In conventional plates, cells experience a relatively uniform oxygen concentration across the media, as gas exchange occurs primarily at the air-liquid interface. However, Seahorse XF plates have a specialized microchamber design that alters the oxygen diffusion dynamics. The sensor cartridge in Seahorse plates temporarily traps a small volume of media over the cells, creating a local microenvironment with fluctuating oxygen tension. These variations in atmospheric versus local oxygen levels at the cell surface can influence mitochondrial respiration, potentially leading to transient metabolic adjustments.

To ensure that metabolic measurements reflect the steady-state phenotype of M0/M1/M2 MDMs, a recovery period of at least 2–4 hours post-reseeding is recommended before initiating the Seahorse assay. This allows cells to stabilize and mitigate stress-induced metabolic fluctuations.

## 5 Pro- and anti-inflammatory molecule secretion by MDMs

When exposed to different stimuli, macrophages rapidly start to secrete pro-inflammatory cytokines, such as Tumor Necrosis Factor (TNF)-α, and interleukins (IL)-1α/β, IL-6 and IL-12. These are critical for activating and amplifying the immune response, or anti-inflammatory cytokines (*i.e.*, IL-10 and transforming growth factor, TGF-β), essential for the resolution of inflammation (138).

Besides cytokines, MDMs can also produce several chemokines aimed at recruiting other immune cells during immune responses. Once differentiated, M0 cells maintain a baseline secretion of chemokines involved in immune surveillance and homeostasis, such as CCL2 (MCP-1; monocyte chemoattractant), CCL3 (MIP-1α; inflammatory monocyte and NK cell recruitment), CCL4 (MIP-1β; macrophage and T-cell recruitment), CCL5 (RANTES; T-cell and eosinophil chemoattractant) and CXCL8 (IL-8; Neutrophil chemoattractant). M1 Mϕ mainly produce chemokines implicated in the recruitment of T-lymphocytes and Natural Killer (NK) cells, such as CXCL9 (MIG), CXCL10 (IP-10), CXCL11 (I-TAC), CCL2 (MCP-1), CCL3 (MIP-1α), CCL4 (MIP-1β), and CCL5, and CXCL8 (IL-8). Finally, M2 Mϕ secrete CCL17 (TARC), CCL18 (MDC), CCL22 (MDC), and CCL24 (Eotaxin-2) to recruit Th2 and T regulatory CD4+ T-cells (**Figure 2**).

Cytokines and chemokines levels can be easily quantified in cell culture supernatants using well-established techniques, such as enzyme-linked immunosorbent assays (ELISA) or Luminex multiplex assays. Despite providing sensitive and specific detection of soluble mediators of inflammation, a key limitation of these methods is their inability to differentiate between increased cytokine output due to either an increase in cell number or an increase in cytokine release per single cell. Alternatively, to capture intracellular cytokine production, cells can be exposed to stimuli for the desired differentiation period and protein transport inhibitors (such as monensin or brefeldin A). These agents block cytokine secretion, allowing the cytokines to accumulate intracellularly. However, prolonged exposure to monensin or brefeldin A is cytotoxic, limiting their use to short-term treatments only. Subsequent cell permeabilization and staining with fluorochrome-conjugated antibodies enable flow cytometric detection of the cytokines. Although this method does not provide an exact quantitation of cytokines, it has the advantage of identifying the proportion of cytokine-producing cells, allowing for the assessment of functional heterogeneity within MDM populations.

Cytokines/chemokines data obtained from ELISA assays or flow cytometry can be complemented by assessing the gene expression levels of key cytokines and chemokines, which can be quantified using real-time quantitative PCR (qRT-PCR). In recent years, digital droplet Polymerase Chain Reaction (ddPCR, Bio-Rad, Hercules, CA, USA) has emerged as a powerful technique for the absolute quantification of gene expression. ddPCR can detect low-abundance targets by partitioning a normal PCR reaction into thousands of droplets, in which fluorescent dye-based end-point PCRs occur independently (139). As such, it provides a robust tool for confirming the expression levels of cytokines and chemokines, especially in experimental settings where subtle differences in gene expression may be biologically significant. However, it is essential to interpret mRNA data with caution, as mRNA levels do not always directly correlate with protein abundance. This discrepancy arises from various post-transcriptional regulatory mechanisms, including mRNA stability, translation efficiency, and protein degradation rates.

M1 MDMs are known to be efficient producers of toxic effector molecules, such as Reactive Oxygen Species (ROS) and Reactive Nitrogen Species (RNS). The term “ROS” includes superoxide anion (O_2_^-^), hydrogen peroxide (H_2_O_2_), hydroxyl radical, and singlet oxygen (140). These reactive molecules are fundamental for pathogen killing through the damage of microbial structure and the activation of the inflammatory signaling pathways. To verify the ability of cells to produce ROS, MDMs can be briefly stimulated with reagents such as hydrogen peroxide (H_2_O_2_) or tert-butyl hydroperoxide (TBHP). ROS production can then be quantified using various assays, including fluorescent probes (such as 2’,7’-dichlorodihydrofluorescein, DCFH) or chemiluminescence, to assess the relative oxidative response (141). However, when quantifying specific ROS, more targeted methods can be employed. For example, the Cytochrome *c* (Cyt*c*) reduction assay is used to directly measure superoxide production, while hydroxyl radical formation can be accessed via HPLC or spectrophotometry, and singlet oxygen levels can be evaluated using the singlet oxygen sensor green.

Besides ROS, M1 Mϕ also generate great amounts of reactive nitrogen species (RNS), a group of small, bioactive molecules that include nitric oxide (NO^-^), hydrogen sulphide, and carbon monoxide. Among these, NO^-^ is particularly noteworthy for its potent bactericidal properties, contributing significantly to the microbicidal function of M1 Mϕ. NO^-^ is synthesized by inducible nitric oxide synthase (iNOS), and the upregulation of iNOS serve as a key indicator of M1 activation, contributing to eliminating pathogens. To assess iNOS expression and NO^-^ production, biochemical techniques such as Western blotting, flow cytometry, and ELISA are commonly used. Additionally, iNOS enzymatic activity can be assessed by quantifying the conversion of L-arginine to L-citrulline. The production of NO^-^ can also be indirectly quantified by measuring nitrite concentrations in the culture medium using the Griess assay, as nitrite is a stable oxidation product of NO^-^. While M1 Mϕ exhibit robust iNOS expression and NO production, alternative (M2) cells, induced by IL-4 and IL-13, typically show low or undetectable levels.

Possible pitfalls:

- Dynamic cytokine profile over time in the differentiated MDMs: Considering the plasticity of Mϕ, their cytokine profiles are highly dynamic and evolve over time, according to the complex regulatory mechanisms that ensure a balanced immune response. Initially, M1 cells are characterized by the rapid production of pro-inflammatory cytokines such as TNF-α, IL-1β, IL-6, and IL-12, which are crucial for pathogen clearance and the activation of adaptive immunity. However, as the inflammatory response progresses, these cells enter a negative feedback loop, characterized by the upregulation of anti-inflammatory cytokines, such as IL-10 (142, 143). This temporal shift represents a physiological mechanism evolved to mitigate excessive and deleterious inflammation, thereby preventing tissue damage and promoting the resolution of inflammation.

To efficiently differentiate monocytes toward an M1 phenotype, cells are typically exposed to a specific cytokine cocktail for a defined (48h) period. Maintaining the M1 under longer cytokine exposure can trigger anti-inflammatory feedback mechanisms, including an increased production of IL-10 (142). The M1 phenotype can be thus compromised by a subsequent shift toward a mixed, or even an anti-inflammatory state. Therefore, precise control throughout cytokine exposure is critical to maximize pro-inflammatory differentiation while minimizing the onset of negative feedback loops. Assessing cytokine profiles at multiple time points is essential for optimizing differentiation protocols and ensuring that macrophages retain their functional characteristics.

- Timing of assessing ROS and RNS production by MDMs: Timing is a critical factor when assessing reactive oxygen (ROS) and reactive nitrogen species (RNS) production in differentiated Mϕ, as their secretion follows distinct kinetic profiles and requires appropriate stimulation. These reactive species have a dynamic and transient secretion pathway. For ROS, peak production usually occurs rapidly, often within minutes following stimulus exposure (such as H_2_O_2_ or TBHP). Therefore, their presence should be assessed promptly, within a window of 10 to 30 minutes post-stimulation, to capture the maximal oxidative response (141).

In contrast, the production of RNS, particularly nitric oxide (NO^-^) synthesized via inducible nitric oxide synthase (iNOS), generally exhibits a delayed kinetic profile due to the time required for iNOS induction and enzyme activation. At the transcriptional level, iNOS mRNA expression can be assessed using RT-qPCR, with a typical peak between 12- and 24-hours post-stimulation.

## 6 Functional abilities of MDMs

The ability of MDMs to functionally reflect their acquired phenotype is an important feature. One of the most fundamental and evolutionarily conserved functions of Mϕ is phagocytosis, a receptor-mediated process essential for the clearance of pathogens, apoptotic cells, and cellular debris. Phagocytosis is primarily mediated by pattern recognition receptors (PRRs) and scavenger receptors, which regulate the uptake of particles larger than 0.5 μm in diameter (144). The efficiency and mechanisms of phagocytosis vary significantly depending on Mϕ polarization. M1 MDMs exhibit the highest phagocytic ability, particularly against microbial pathogens, owing to their upregulation of opsonic and non-opsonic receptors, such as Fcγ receptors and complement receptors (120). The enhanced phagocytic activity of this phenotype, coupled with the production of ROS and RNS, promotes pathogen engulfment and intracellular killing. In contrast, M2 MDMs exhibit reduced phagocytic efficiency but display increased endocytic activity, in accordance with their role in facilitating dying cells clearance and the resolution of inflammation. This process is mediated by scavenger receptors such as CD163 and CD206, which facilitate the engulfment of modified lipoproteins and cellular debris. M0 MDMs, representing the unpolarized state, retain a baseline level of phagocytic activity but lack the specialized enhancements described for M1 or M2 subsets (17).

**Figure 3:**
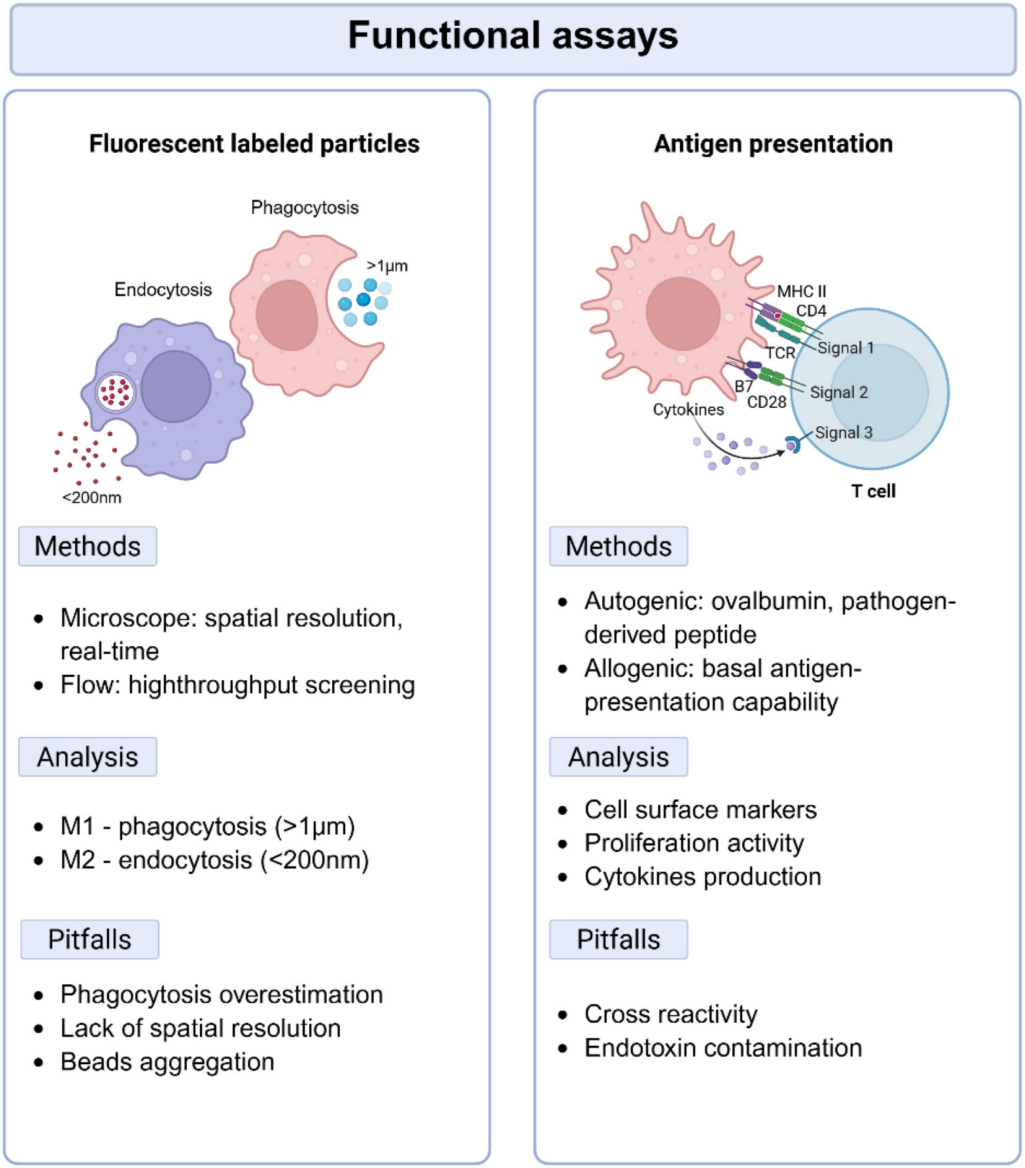
Overview of functional assays to evaluate macrophage activity. (Left) Uptake assays using fluorescently labelled particles to detect the phagocytic ability of MDMs. The figure outlines different methods to detect it (e.g., flow cytometry, microscopy) and critical pitfalls. (Right) Antigen presentation assays used to evaluate MDMs interaction with T-cells, including co-culture systems with antigenspecific CD4^+^ or CD8^+^ T cells. Various readouts are shown alongside challenges.

Several methods have been developed to measure phagocytic or endocytic activity, depending on the experimental design and the desired level of sensitivity and resolution (**Figure 3**). The most employed technique relies on the use of microscopy. Cells are incubated with commercially available fluorescently labelled particles, typically larger beads (*e.g.*, >1 µm) for phagocytosis and smaller beads (*e.g.*, <200 nm) for endocytosis. The larger particles are often conjugated with PAMPs obtained from *E. coli*, *S. aureus*, or Zymosan (yeast cell wall extract), which engage PRRs and are preferentially engulfed by pro-inflammatory M1 MDMs. In contrast, smaller dextran beads enter cells via receptor-mediated endocytosis, a process typical of M2 cells. The internalized material is then observed using fluorescence microscopy. Quantification is done by counting the number of cells that have internalized the particles or by measuring the fluorescence intensity associated with phagocytic activity (145).

Alternatively, flow cytometry can be employed for both phagocytosis and endocytosis. This technique offers the advantage of rapidly assessing the uptake of particles across a larger number of cells, providing a high-throughput approach to particle uptake that cannot be matched by fluorescent microscopy. In contrast, fluorescence microscopy not only provides spatial information but also allows real-time visualization of live cells actively performing phagocytosis or endocytosis, offering valuable insights into uptake dynamics.

Another key function of Mϕ, as APCs, is the presentation of antigens to CD4^+^ T-lymphocytes (5). Among the different MDMs phenotypes, M1 cells are the most efficient in antigen presentation, largely due to their high expression of MHC-II and co-stimulatory molecules (*e.g.*, CD80, CD86, and CD40) compared to M0 and M2 MDMs (120). The expression levels of these molecules are commonly considered an indirect indicator of the antigen-presenting ability of MDMs.

Alternatively, antigen presentation capacity can also be directly assessed using functional assays (**Figure 3**). MDMs can be pulsed with a known antigen, such as pathogen-derived peptides, and then co-cultured with antigen-specific CD4^+^ T-cells, typically isolated from the same donor (146, 147). The ability of MDMs to present the antigens can be assessed by measuring T-cell activation as output, which is commonly evaluated by measuring the upregulation of activation markers, such as CD69, CD25, 41BB, and CD40L, using flow cytometry (148). These markers are typically upregulated following T-cell receptor engagement with antigen-MHC-II complexes on the MDMs surface, indicating successful antigen recognition and initiation of T-cell activation.

Following activation, T-cells undergo clonal expansion, a process that can be detected by flow cytometry by measuring carboxyfluorescein succinimidyl ester (CFSE) dilution, which tracks T-cell division (146, 147). Proliferation is driven by T-cell receptor (TCR) signaling cascade, which induces clonal expansion to generate additional T-cells capable of recognizing the same antigen. Furthermore, the production of pro-inflammatory cytokines, such as IFN-γ, IL-2, and TNF-α, can be quantified using assays like ELISA, ELISPOT, or intracellular cytokine staining. Cytokine secretion is a key downstream event triggered by the activation of transcription factors like NF-κB and AP-1, which drive the expression of genes involved in immune responses, including those that support T-cell differentiation, Mϕ activation, and the orchestration of the adaptive immune response.

Possible pitfalls.

- Overestimation of phagocytosis by flow cytometry: A key limitation of flow cytometry is its inability to distinguish between surface-bound and fully internalized particles, which can lead to an overestimation of particle uptake. While it provides quantitative data, it lacks the spatial details needed to determine the localization of particles within the cell. Moreover, fluorescent beads or bacterial particles may aggregate, potentially generating artificially high fluorescence signals that do not accurately reflect individual cell uptake.

To mitigate these issues, it is crucial to thoroughly resuspend the beads and perform frequent washing steps throughout the experiment to remove any uninternalized particles. This approach helps eliminate non-specific binding and ensures that only particles actively engulfed by MDMs are counted in the analyses. Additionally, incorporating live/dead staining can be highly advantageous, as it allows for the exclusion of sticky aggregates composed of dead cells and beads.

- Non-specific Activation of T-cells: In functional assays, non-specific activation of T-cells (*e.g.*, due to cross-reactivity or contamination with endotoxins) may lead to background signals. To control such effects, it is important to assess the baseline activation, proliferation, and cytokine production of CD4^+^ T-cells cultured in the absence of MDMs. The specificity of activation due to the MHC-II can also be verified through the inclusion of MHC-II blocking antibodies which inhibit antigen presentation and thereby verify that observed T-cell responses are specifically mediated via MHC-II–restricted pathways.

## 7 Pros and cons in the use of MDMs and future perspectives

MDMs offer a robust and physiologically relevant human-based *in vitro* model for studying Mϕ biology, their interactions with other cell types, immune responses, and host-pathogen interactions. In contrast to immortalized cell lines, such as THP-1 (149), or U-937 (150), MDMs offer a more physiologically relevant model, as they are primary cells better reflecting the functional and transcriptional diversity of human Mϕ.

Although it is well established that TRMs are important in health and disease, the contribution of MDMs to the tissue macrophage population in homeostasis and disease increases over time. It strongly affects the course and outcome of subsequent inflammation, immune activation, and disease development, thereby underscoring the importance of MDM-based models to dissect macrophage biology and develop novel therapeutic strategies.

Compared to TRMs, MDMs can be obtained from peripheral blood monocytes, which can be collected through minimally invasive procedures such as routine blood draws or donation, avoiding the need for surgical biopsies to access specific tissues. The feasibility of collection not only reduces patient burden and ethical concerns but also allows repeated sampling from the same individual over time, facilitating longitudinal (time-resolved) studies of immune responses. Since MDMs can be derived from both healthy donors and patients, they provide a versatile platform for both disease-focused and basic research. Cells from healthy individuals enable researchers to investigate baseline Mϕ biology, to dissect the molecular mechanisms underlying polarization and immune activation. At the same time, patient-derived MDMs capture disease-specific features, allowing the study of immune dysfunctions associated with genetic variants, infections, or inflammatory conditions. Thus, the use of these cells facilitates comparative studies between patient-derived and control MDMs, offering insights into altered Mϕ function in pathological contexts.

To further contextualize MDMs within the broader macrophage landscape, a comparative analysis of both phenotypic/functional parameters between MDM-derived subsets (M0, M1, M2, and alveolar-like) and primary TRMs should be always considered. This comparison must integrate multiple layers of macrophage biology, encompassing transcriptional signatures, effector functions, and functional plasticity. For instance, TRMs exhibit enrichment of tissue-specific genes such as PPARG, MARCO, and CSF2RA in alveolar macrophages, reflecting stable programming driven by local microenvironmental cues. Functionally, MDMs display robust phagocytic and endocytic capacities that closely model core macrophage programs, while TRMs maintain specialized endocytic preferences and tightly regulated cytokine secretion patterns tailored to their tissue of residence. Cytokine profiling further demonstrates that pro-inflammatory M1-like MDMs secrete high levels of TNF-α and IL-1β, whereas M2-like MDMs produce IL-10 and chemokines associated with tissue repair. TRMs, in contrast, show context-dependent responses shaped by chronic microenvironmental exposure, maintaining a balanced basal activation state.

Moreover, MDMs offer flexibility in experimental design. They can be polarized into distinct functional phenotypes (M0, M1, M2 and TRMs) and study their responses to various stimuli, making them important tools for understanding key immunological pathways and disease mechanisms, particularly in viral and bacterial infectious diseases, inflammatory disorders, and cancer.

While cell lines provide a convenient and reproducible system due to their uniformity and ease of culture, they often exhibit altered signaling pathways, reduced plasticity, and genetic drift from prolonged passaging, which can limit their ability to accurately mimic *in vivo* Mϕ behavior. In contrast, MDMs retain donor-specific immune characteristics, making them particularly useful for studying inter-individual variability in immune responses. However, this variability is also one of the primary challenges for experimental reproducibility in their use (151). Nonetheless, this limitation can be effectively overcome by increasing the number of donors in a study, ensuring a more representative and statistically robust dataset while preserving the physiological relevance of primary human Mϕ.

It is important to underline that MDMs lack long-term exposure to the complex microenvironmental cues present in the tissues that shape TRMs identity *in vivo*, including cytokine gradients, cellular interactions, and metabolic conditions specific to tissues such as the lung, liver, or gut (99). This makes it difficult to fully recapitulate the functional diversity of Mϕ subsets found in different organs.

To address this limitation, various differentiation protocols incorporating specific cytokines and growth factors have been tested to generate MDMs with specialized phenotypes that closely resemble TRMs or those found in specific disease contexts, as previously described (100, 152).

TRMs have also been recently differentiated *in vitro* from induced pluripotent stem cells (iPSCs) obtained from stromal cells or embryonic stem cells, providing a promising complementary approach to MDM-based models (153). Successful examples include the generation of iPSC-derived microglia through cocktails of M-CSF, IL-34, TGF-β, CD200, and CX3CL1, which displayed high transcriptomic similarity to primary microglia and faithfully recapitulated disease-relevant phenotypes, such as TREM2-related deficits in phagocytosis (154, 155). Similar approaches have been applied to model pulmonary alveolar proteinosis and Gaucher disease (156), as well as to generate macrophages carrying engineered mutations using CRISPR/Cas9 to dissect the role of genes such as ABCA1 or LIPA (lipase A) in lipid metabolism and inflammation (157). These findings highlight the potential of iPSDMs to overcome some of the limitations of MDMs, such as the restricted ability to acquire stable tissue-resident identities and the difficulties in introducing targeted mutations into their genomes (158).

Collectively, these approaches underscore the plasticity of MDMs and demonstrate that strategic modulation of the differentiation environment enables the generation of Mϕ subtypes that recapitulate key features of tissue-resident or disease-associated Mϕ. Furthermore, the ability to manipulate macrophage differentiation opens new avenues for therapeutic interventions aimed at reprogramming Mϕ in disease settings.

Looking at the future, a key challenge will be the development and rigorous standardization of differentiation protocols able to faithfully recapitulate tissue-specific Mϕ identities without altering experimental reproducibility. Integration of MDM-based models with the use of cutting-edge technologies, such as single-cell transcriptomics, organ-on-chip systems, and 3D co-cultures, will likely enhance their physiological fidelity and predictive power. Indeed, while Mϕs’ biology has been extensively studied in traditional two-dimensional (2D) culture systems, yet it is evident that such models fail to fully capture critical aspects of their *in vivo* counterpart (159). There is emerging evidence of additional tissue-specific properties critically modulate macrophage phenotype and behavior. These include the extracellular matrix (ECM) composition of tissues and its interaction with macrophage integrin receptors and other proteins, the mechanical properties of tissues such as stiffness or elasticity, the presence and cross-talk of neighboring cell types, and the concentration of small molecules and metabolites (160). This is particularly relevant in the context of the tumor microenvironment, where 3D culture models better recapitulate spatial and mechanical influences that shape macrophage activation states and functional heterogeneity (161, 162). Within this context, the ECM does not simply provide structural support but also exerts functional control over macrophage activity. For instance, Mϕs cultured on stiffer substrates produce higher amounts of pro-inflammatory cytokines in response to LPS compared to those maintained on softer matrices (163). Beyond these biophysical inputs, intercellular communication further dictates macrophage responses. Crosstalk with stromal cells, such as fibroblasts, modulates growth factor exchange, prevents aberrant proliferation, and orchestrates tissue remodeling, thereby integrating Mϕs into broader network (164). Adding another layer of regulation, extracellular vesicles and exosomes have emerged as key mediators of macrophage–stromal and macrophage–immune cell interactions, enabling communication within tissues (165). Collectively, these insights highlight the need to move beyond oversimplified 2D models toward standardized, multidimensional systems that more accurately capture the complexity of tissue microenvironments in which macrophages operate in both health and disease.

Moreover, leveraging patient-derived MDMs in personalized medicine frameworks may accelerate the discovery of biomarkers and the development of therapeutic precision therapeutics tailored to individual immune landscapes, including those associated with rare and complex diseases. Ultimately, while MDMs cannot fully replace *in vivo* or tissue-resident models, their ongoing methodological refinements hold great promise for bridging the gap between basic research and clinical translation in diverse fields such as infections and cancer.

Looking forward, advancing MDM research will depend on bridging the gap between simplified *in vitro* models and the complex functional states of tissue-resident macrophages. While TRMs provide tissue-specific insights, their limited availability and difficulty in isolation constrain their experimental use. Standardized MDMs offer a scalable and controllable platform, but achieving reproducible results requires careful attention to monocyte isolation, cytokine exposure, metabolic conditions, and prevention of contamination. Future strategies should focus on integrating MDMs with physiologically relevant systems, including 3D organoid co-cultures, organ-on-chip platforms, and extracellular matrix–mimicking scaffolds, to better replicate the spatial, mechanical, and biochemical cues present in tissues. Coupling these approaches with single-cell and spatial transcriptomic analyses will allow high-resolution characterization of macrophage heterogeneity and functional plasticity. This combination will enable patient-specific modeling, investigation of inter-individual variability, and development of macrophage-targeted therapies. By uniting standardized MDM protocols with multidimensional, tissue-relevant systems, researchers can generate more predictive and translational insights into macrophage biology in both health and disease.
